# Supplementary material for: Hot Water Extraction of Antioxidants from Tea Leaves—Optimization of Brewing Conditions for Preparing Antioxidant-Rich Tea Drinks
Source: Molecules. 2023 Mar 28;28(7):3030. doi: 10.3390/molecules28073030 (PMC10095724; doi:10.3390/molecules28073030)
Supplement: Supplementary file 1 [file molecules-28-03030-s001.zip › molecules-2291558-supplementary.pdf]

Supplementary results

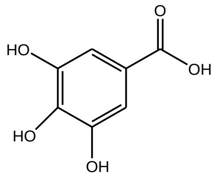
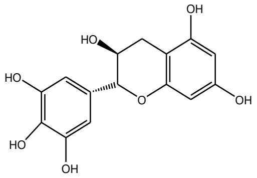
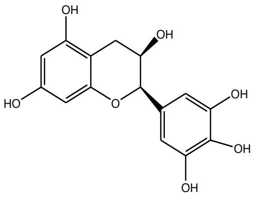
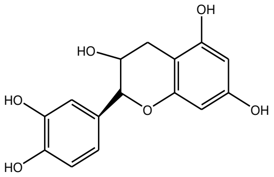

Gallic acid (+)-Gallocatechin (-)-Epigallocatechin Cianidanol

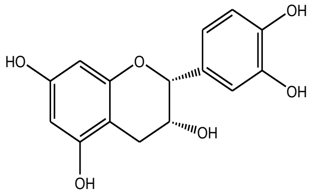
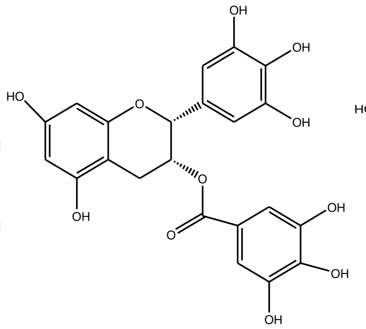
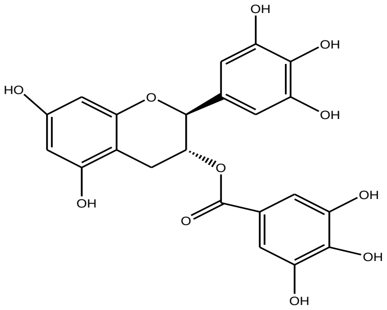

L-Epicatechin (-)-Epigallocatechin gallate (-)-Gallocatechin gallate

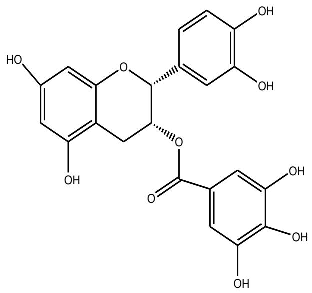
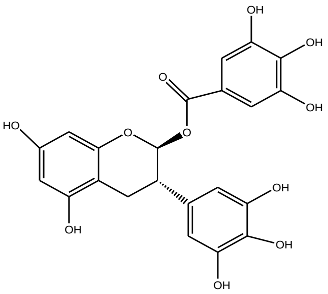
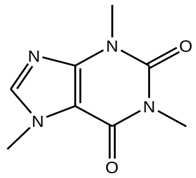

(-)-Epicatechin gallate Catechin gallate Caffeine

Figure S1. Structures of polyphenols and caffeine.

Table S1. Kinetic effect on antioxidant concentrations with 20-min brewing time.

Temp. (°C)	mg/g of Rizhao Tea **									
	GA*	GC	EGC	C	CAF	EC	EGCG	GCG	ECG	CG
25	0.16 ± 0.04**	1.22 ± 0.13	7.58 ± 0.75	0.88 ± 0.13	4.97 ± 0.38	4.65 ± 0.17	3.36 ± 0.53	0.41 ± 0.19	0.25 ± 0.10	0.27 ± 0.09
50	0.32 ± 0.08	3.46 ± 0.47	11.55 ± 0.12	1.01 ± 0.28	12.02 ± 0.16	4.85 ± 0.12	7.37 ± 0.18	0.53 ± 0.08	1.47 ± 0.35	0.30 ± 0.02
70	0.39 ± 0.10	4.04 ± 0.26	17.13 ± 0.16	1.04 ± 0.15	17.62 ± 0.15	4.91 ± 0.70	9.96 ± 0.26	0.55 ± 0.01	2.09 ± 0.27	0.34 ± 0.17
80	0.43 ± 0.08	4.22 ± 0.35	19.22 ± 0.17	1.20 ± 0.09	19.69 ± 0.10	5.20 ± 0.41	12.76 ± 0.15	0.61 ± 0.14	2.20 ± 0.30	0.37 ± 0.07
90	0.45 ± 0.23	4.31 ± 0.57	22.21 ± 0.37	1.49 ± 0.19	20.33 ± 0.13	6.04 ± 0.80	15.97 ± 0.12	0.79 ± 0.24	2.93 ± 0.44	0.41 ± 0.10
100	0.48 ± 0.05	4.40 ± 0.55	26.88 ± 0.31	1.62 ± 0.06	20.44 ± 0.15	6.53 ± 0.89	18.80 ± 0.87	0.92 ± 0.17	3.42 ± 0.33	0.49 ± 0.02
	mg/g of Longjing Tea									
25	0.39 ± 0.19	0.49 ± 0.22	8.98 ± 0.10	0.84 ± 0.17	7.40 ± 0.65	1.57 ± 0.36	9.30 ± 0.25	0.19 ± 0.10	1.27 ± 0.51	0.50 ± 0.05
50	0.61 ± 0.16	0.75 ± 0.39	15.82 ± 0.39	1.47 ± 0.74	10.87 ± 0.18	2.74 ± 0.73	15.04 ± 0.18	0.30 ± 0.14	2.64 ± 0.29	0.61 ± 0.09
70	0.99 ± 0.11	0.89 ± 0.78	16.46 ± 0.37	1.51 ± 0.08	14.49 ± 0.21	4.71 ± 0.11	16.53 ± 0.33	0.36 ± 0.09	3.05 ± 0.45	0.60 ± 0.40

80	1.15 ± 0.15	0.95 ± 0.78	18.46 ± 0.33	1.75 ± 0.28	16.18 ± 0.80	6.33 ± 0.73	18.86 ± 0.11	0.39 ± 0.12	3.25 ± 0.66	0.69 ± 0.37
90	1.29 ± 0.10	1.68 ± 0.67	20.93 ± 0.10	2.29 ± 0.58	16.79 ± 0.16	7.96 ± 0.99	21.17 ± 0.37	0.40 ± 0.09	3.86 ± 0.47	0.75 ± 0.15
100	1.49 ± 0.26	2.32 ± 0.66	26.60 ± 0.20	2.83 ± 0.62	17.86 ± 0.16	8.25 ± 0.28	21.75 ± 0.34	0.41 ± 0.15	3.96 ± 0.12	0.77 ± 0.22
mg/g of Tieguanyin Tea										
25	0.50 ± 0.35	0.76 ± 0.11	1.83 ± 0.16	0.49 ± 0.10	3.32 ± 0.82	1.17 ± 0.87	2.86 ± 0.40	0.09 ± 0.09	0.48 ± 0.13	0.26 ± 0.23
50	1.05 ± 0.17	1.24 ± 0.73	5.35 ± 0.28	0.82 ± 0.62	5.19 ± 0.40	2.08 ± 0.47	5.01 ± 0.11	0.21 ± 0.15	0.88 ± 0.33	0.51 ± 0.11
70	1.33 ± 0.09	1.96 ± 0.33	13.93 ± 0.19	1.13 ± 0.36	10.91 ± 0.23	2.40 ± 0.09	10.09 ± 0.29	0.34 ± 0.12	1.71 ± 0.47	0.74 ± 0.12
80	1.40 ± 0.21	1.99 ± 0.02	14.50 ± 0.12	1.28 ± 0.05	12.96 ± 0.45	2.58 ± 0.22	11.62 ± 0.19	0.39 ± 0.10	2.13 ± 0.04	0.82 ± 0.04
90	1.54 ± 0.18	3.22 ± 0.66	16.58 ± 0.11	1.33 ± 0.30	15.12 ± 0.13	2.63 ± 0.48	13.00 ± 0.15	0.42 ± 0.07	3.01 ± 0.22	0.88 ± 0.20
100	1.57 ± 0.42	3.47 ± 0.85	16.67 ± 0.35	1.41 ± 0.23	16.71 ± 0.17	2.84 ± 0.86	13.98 ± 0.14	0.45 ± 0.11	3.04 ± 0.25	0.90 ± 0.07
mg/g of Dahongpao Tea										
25	0.06 ± 0.04	0.91 ± 0.16	5.49 ± 0.82	0.43 ± 0.08	1.49 ± 0.18	1.07 ± 0.18	2.29 ± 0.62	ND***	0.44 ± 0.14	ND
50	0.13 ± 0.08	1.27 ± 0.58	6.32 ± 0.20	0.59 ± 0.10	3.12 ± 0.36	1.41 ± 0.28	5.89 ± 0.66	ND	0.85 ± 0.77	ND
70	0.16 ± 0.09	1.53 ± 0.59	14.23 ± 0.12	0.63 ± 0.08	5.11 ± 0.95	2.45 ± 0.19	8.52 ± 0.40	ND	1.20 ± 0.44	ND
80	0.17 ± 0.04	1.65 ± 0.16	14.72 ± 0.37	0.68 ± 0.03	5.58 ± 0.89	2.67 ± 0.31	9.05 ± 0.85	0.08 ± 0.04	1.57 ± 0.26	ND
90	0.19 ± 0.05	1.73 ± 0.55	15.58 ± 0.11	0.69 ± 0.04	5.72 ± 0.97	2.80 ± 0.69	10.58 ± 0.18	0.13 ± 0.01	1.65 ± 0.20	0.10 ± 0.02
100	0.19 ± 0.10	1.80 ± 0.18	18.20 ± 0.24	0.70 ± 0.17	7.04 ± 0.73	2.93 ± 0.07	11.16 ± 0.69	0.14 ± 0.03	1.75 ± 0.12	0.14 ± 0.01
mg/g of Pu-erh Tea										
25	0.10 ± 0.02	ND	ND	ND	ND	ND	ND	ND	ND	ND
50	0.25 ± 0.07	ND	ND	ND	2.31 ± 0.56	ND	ND	ND	ND	ND
70	0.36 ± 0.23	ND	ND	ND	5.04 ± 0.16	ND	ND	ND	ND	ND
80	0.58 ± 0.12	ND	ND	ND	5.41 ± 0.17	ND	ND	ND	ND	ND
90	0.74 ± 0.26	ND	ND	ND	7.01 ± 0.11	ND	ND	ND	ND	ND
100	0.75 ± 0.19	ND	ND	ND	7.19 ± 0.85	ND	ND	ND	ND	ND
mg/g of Zhengshanxiaozhong Tea										
25	0.21 ± 0.02	ND	ND	ND	ND	ND	ND	ND	ND	ND
50	0.39 ± 0.06	0.44 ± 0.24	1.87 ± 0.98	0.90 ± 0.04	4.87 ± 0.40	1.15 ± 0.15	1.60 ± 0.11	ND	ND	ND
70	0.45 ± 0.14	1.06 ± 0.24	3.44 ± 0.28	0.97 ± 0.08	8.39± 0.43	2.08 ± 0.30	3.32 ± 0.03	ND	0.24 ± 0.06	ND
80	0.65 ± 0.14	1.68 ± 0.44	3.92 ± 0.26	1.92 ± 0.17	10.30 ± 0.96	2.36 ± 0.47	4.01 ± 0.34	ND	0.46 ± 0.09	ND
90	0.76 ± 0.17	3.00 ± 0.04	4.43 ± 0.56	2.14 ± 0.15	11.15 ± 1.01	2.70 ± 0.30	4.13 ± 0.10	ND	0.49 ± 0.10	ND
100	1.55 ± 0.10	5.35 ± 0.70	4.77 ± 0.67	2.75 ± 0.14	12.02 ± 1.08	4.54 ± 0.05	4.32 ± 0.01	ND	0.51 ± 0.08	ND
mg/g of Jasmine Tea										
25	0.08 ± 0.02	0.20 ± 0.05	0.74 ± 0.18	ND	1.26 ± 0.31	0.16 ± 0.05	0.15 ± 0.04	ND	0.05 ± 0.01	ND
50	0.16 ± 0.13	0.35 ± 0.04	1.49 ± 0.08	0.16 ± 0.01	2.65 ± 0.73	0.32 ± 0.09	0.39 ± 0.07	ND	0.08 ± 0.04	ND
70	0.49 ± 0.24	0.68 ± 0.05	3.41 ± 0.13	0.35 ± 0.07	6.36 ± 0.24	0.50 ± 0.09	0.96 ± 0.11	ND	0.21 ± 0.02	0.11 ± 0.03
80	0.53 ± 0.15	1.40 ± 0.16	5.49 ± 0.09	0.59 ± 0.09	9.49 ± 1.64	1.03 ± 0.07	1.57 ± 0.34	0.08 ± 0.02	0.36 ± 0.02	0.25 ± 0.09
90	0.94 ± 0.27	1.60 ± 0.10	6.42 ± 0.10	0.76 ± 0.07	11.94 ± 0.83	1.62 ± 0.10	1.89 ± 0.21	0.15 ± 0.03	0.42 ± 0.06	0.40± 0.08
100	1.02 ± 0.08	1.67 ± 0.25	6.81 ± 0.11	0.82 ± 0.08	12.68 ± 0.32	1.81 ± 0.05	2.10 ± 0.07	0.21 ± 0.07	0.44 ± 0.05	0.45 ± 0.09

* Gallic acid (GA), (+)-Gallocatechin (GC), (-)-Epigallocatechin (EGC), Cianidanol (C), Caffeine (CAF), L-Epicatechin (EC), (-)-Epigallocatechin gallate (EGCG), (-)-Gallocatechin gallate (GCG), (-)-Epicatechin gallate (ECG), Catechin gallate (CG)

** Mean (± SD), n = 3

***ND, not detected, below the detection limit shown in Table 2.

Table S2. Kinetic effect on antioxidant concentrations at 100 °C.

Time (min)			mg/g of Rizhao Tea**							
	GA*	GC	EGC	C	CAF	EC	EGCG	GCG	ECG	CG
5	0.40 ± 0.06**	2.40± 2.08	15.95 ± 2.34	0.50 ± 0.05	17.57 ± 2.29	3.36 ± 0.67	6.17 ± 1.59	0.24 ± 0.06	0.79 ± 0.29	0.30 ± 0.04
10	0.43 ± 0.05	3.10± 0.55	21.88 ± 0.31	1.02 ± 0.06	18.44 ± 0.15	5.63 ± 0.89	9.84 ± 0.87	0.78 ± 0.17	2.60 ± 0.33	0.41 ± 0.02
20	0.48 ± 0.05	4.40 ± 0.55	26.88 ± 0.31	1.62 ± 0.06	20.44 ± 0.15	6.53 ± 0.89	18.80 ± 0.87	0.92 ± 0.17	3.42 ± 0.33	0.49 ± 0.02
30	0.55 ± 0.06	5.47 ± 0.10	30.14 ± 0.33	2.10 ± 0.05	22.12 ± 1.19	8.29 ± 0.67	23.72 ± 3.12	1.06 ± 0.18	4.46 ± 0.36	0.58 ± 0.12
60	0.65 ± 0.03	6.27 ± 0.12	35.12 ± 0.30	3.03 ± 0.21	22.97 ± 1.05	10.88 ± 0.38	29.87 ± 2.30	1.29 ± 0.15	5.80 ± 0.47	0.77 ± 0.05
120	0.75 ± 0.24	6.22 ± 0.07	40.12 ± 0.28	3.56 ± 0.10	23.27 ± 0.58	11.98 ± 0.39	32.25 ± 2.21	1.31 ± 0.15	6.33 ± 0.37	0.85 ± 0.06
720	0.88 ± 0.39	3.63 ± 3.81	47.47 ± 0.38	3.02 ± 0.14	23.91 ± 0.51	3.22 ± 0.17	3.55 ± 0.32	1.01 ± 0.06	5.53 ± 0.55	0.20 ± 0.02
					mg/g of Longjing Tea					
5	0.81 ± 0.21	1.25 ± 0.68	11.70 ± 1.42	1.05 ± 0.10	11.25 ± 4.56	3.38 ± 1.34	6.96 ± 0.37	ND***	0.98 ± 0.79	0.34 ± 0.03
10	1.23 ± 0.03	1.89 ± 0.76	19.88 ± 1.93	2.05 ± 0.12	15.16 ± 2.58	5.66 ± 0.53	10.72 ± 0.50	0.09 ± 0.08	2.78 ± 0.65	0.60 ± 0.02
20	1.49 ± 0.26	2.32 ± 0.66	26.60 ± 0.20	2.83 ± 0.62	17.86 ± 0.16	8.25 ± 0.28	21.75 ± 0.34	0.41 ± 0.15	3.96 ± 0.12	0.77 ± 0.22
30	1.67 ± 0.33	2.84 ± 1.20	31.40 ± 5.58	3.89 ± 0.29	19.34 ± 3.31	10.06 ± 2.13	32.61 ± 1.23	0.64 ± 0.18	5.66 ± 1.16	0.92 ± 0.04
60	1.91 ± 0.17	4.33 ± 1.06	36.09 ± 3.67	5.06 ± 0.29	21.39 ± 1.73	11.69 ± 0.93	37.99 ± 2.02	0.73 ± 0.07	6.67 ± 0.60	1.09 ± 0.04
120	2.15 ± 0.31	1.50 ± 0.54	39.71 ± 1.94	4.87 ± 0.20	21.59 ± 1.45	12.10 ± 0.96	39.41 ± 1.55	0.95 ± 0.06	7.06 ± 0.46	1.15 ± 0.04
720	2.25 ± 0.08	0.28 ± 1.15	44.19 ± 2.62	0.49 ± 0.18	22.11 ± 0.16	1.28 ± 0.11	3.79 ± 0.22	0.10 ± 0.05	0.75 ± 1.46	0.07 ± 0.04
					mg/g of Tieguanyin Tea					
5	0.81 ± 0.04	2.62 ± 0.32	14.66 ± 0.75	1.15 ± 0.04	15.46 ± 1.16	2.36 ± 0.01	11.86 ± 0.89	0.36 ± 0.04	2.21 ± 0.11	0.75 ± 0.07
10	1.20 ± 0.07	2.93 ± 0.14	15.50 ± 0.79	1.23 ± 0.05	15.81 ± 0.97	2.65 ± 0.01	12.67 ± 0.83	0.38 ± 0.04	2.34 ± 0.12	0.82 ± 0.07
20	1.57 ± 0.42	3.47 ± 0.85	16.67 ± 0.35	1.41 ± 0.23	16.71 ± 0.17	2.84 ± 0.86	13.98 ± 0.14	0.45 ± 0.11	3.04 ± 0.25	0.90 ± 0.07
30	1.86 ± 0.11	3.94 ± 1.22	17.32 ± 0.73	1.54 ± 0.07	17.14 ± 0.85	3.02 ± 0.01	14.47 ± 0.80	0.50 ± 0.04	3.48 ± 0.15	0.95 ± 0.08
60	2.25 ± 0.45	4.02 ± 2.23	18.53 ± 1.03	1.74 ± 0.20	18.30 ± 0.87	3.31 ± 0.10	15.99 ± 1.00	0.65 ± 0.01	4.35 ± 0.19	1.02 ± 0.12
120	2.56 ± 0.18	3.26 ± 0.51	18.56 ± 0.89	1.78 ± 0.11	20.81 ± 1.57	2.43 ± 0.02	19.15 ± 1.21	0.81 ± 0.13	5.00 ± 0.95	0.43 ± 0.22
720	2.69 ± 0	0.96 ± 0.05	18.93 ± 0.21	0	21.21 ± 0.04	0.71 ± 0.06	1.81 ± 0.07	ND	0.19 ± 0.01	ND
					mg/g of Dahongpao Tea					
5	0.06 ± 0.05	0.89 ± 0.15	8.60 ± 2.10	0.33 ± 0.09	3.72 ± 0.56	1.37 ± 0.13	5.69 ± 0.21	0.08 ± 0.05	0.83 ± 0.24	0.04 ± 0
10	0.11 ± 0.02	1.29 ± 0.15	12.87 ± 2.19	0.46 ± 0.18	5.21 ± 0.81	2.03 ± 0.21	8.56 ± 0.81	0.11 ± 0.09	1.25 ± 0.25	0.09 ± 0.01
20	0.19 ± 0.10	1.80 ± 0.18	18.20 ± 0.24	0.70 ± 0.17	7.04 ± 0.73	2.93 ± 0.07	11.16 ± 0.69	0.14 ± 0.03	1.75 ± 0.12	0.14 ± 0.01
30	0.25 ± 0.01	2.41 ± 0.13	22.98 ± 3.36	0.91 ± 0.10	8.06 ± 0.59	3.86 ± 0.37	13.95 ± 0.58	0.17 ± 0.01	2.21 ± 0.32	0.18 ± 0.01
60	0.34 ± 0.15	3.96 ± 0.06	31.09 ± 3.29	1.21 ± 0.10	9.92 ± 0.22	5.22 ± 0.19	17.72 ± 0.28	0.18 ± 0.05	2.67 ± 0.18	0.24 ± 0.01
120	0.45 ± 0.02	3.23 ± 0.27	37.33 ± 5.40	1.50 ± 0.17	11.79 ± 0.66	6.49 ± 0.50	21.01 ± 0.47	0.21 ± 0.04	3.13 ± 0.34	0.21 ± 0
720	0.51 ± 0.06	1.27 ± 0.71	38.36 ± 4.45	1.43 ± 0.11	14.88 ± 1.33	4.98 ± 0.32	3.48 ± 0.18	0.19 ± 0.09	0.56 ± 0.28	0.20 ± 0
					mg/g of Pu-erh Tea					
5	0.35 ± 0.08	ND	ND	ND	3.21 ± 0.52	ND	ND	ND	ND	ND
10	0.59 ± 0.17	ND	ND	ND	5.16 ± 0.20	ND	ND	ND	ND	ND
20	0.75 ± 0.19	ND	ND	ND	7.19 ± 0.85	ND	ND	ND	ND	ND
30	0.90 ± 0.16	ND	ND	ND	8.93 ± 0.08	ND	ND	ND	ND	ND
60	1.45 ± 0.64	ND	0.25 ± 0.44	ND	13.69 ± 0.43	ND	ND	ND	ND	ND
120	2.83 ± 0.42	ND	0.88 ± 0.06	ND	22.83 ± 0.81	ND	ND	ND	ND	ND
720	3.52 ± 0.15	ND	1.24 ± 1.09	ND	26.02 ± 0.58	ND	ND	ND	ND	ND
					mg/g of Zhengshanxiaozhong Tea					
5	0.60 ± 0.02	1.56 ± 0.18	1.70± 0.29	0.35 ± 0.04	4.82 ± 0.83	1.18 ± 0.04	1.83 ± 0.55	ND	0.30 ± 0.03	ND
10	1.07 ± 0.35	3.05 ± 0.67	3.38 ± 0.98	1.62 ± 0.13	9.02 ± 0.33	3.00 ± 0.11	3.17 ± 0.39	ND	0.42 ± 0.12	ND
20	1.55 ± 0.10	5.35 ± 0.70	4.77 ± 0.67	2.75 ± 0.14	12.02 ± 1.08	4.54 ± 0.05	4.32 ± 0.01	ND	0.51 ± 0.08	ND
30	1.92 ± 1.37	6.75 ± 0.64	5.84 ± 0.47	3.89 ± 0.14	15.90 ± 0.76	8.73 ± 0.19	7.13 ± 0.11	0.45± 0.48	0.67 ± 0.31	0.12 ± 0.01
60	2.21 ± 0.23	7.72 ± 0.39	6.34 ± 0.26	5.10 ± 0.05	16.20 ± 0.28	14.11 ± 0.45	9.37 ± 0.39	0.72± 0.13	1.12 ± 0.16	0.21 ± 0.02
120	2.60 ± 0.48	6.24 ± 0.16	6.57 ± 0.25	5.61 ± 0.06	23.71 ± 0.20	15.49 ± 0.56	16.54 ± 0.23	1.08 ± 0.08	1.25 ± 0.09	0.24 ± 0
720	4.47 ± 0.30	3.33 ± 0.24	7.02 ± 0.11	0.38 ± 0.01	24.65 ± 0.29	2.79 ± 0.14	3.73 ± 0.05	0.17 ± 0.29	0.40 ± 0.01	0.09 ± 0.02
					mg/g of Jasmine Tea					
5	0.87 ± 0.07	1.09 ± 0.21	3.45 ± 0.35	0.58 ± 0.05	3.70 ± 0.07	1.24 ± 0.17	1.53 ± 0.12	0.15 ± 0.03	0.20 ± 0.02	0.27 ± 0.04
10	0.91 ± 0.15	1.38 ± 0.39	5.01 ± 1.08	0.66 ± 0.04	8.90 ± 0.06	1.56 ± 0.29	1.78 ± 0.14	0.19 ± 0.03	0.35 ± 0.05	0.32 ± 0.02
20	1.02 ± 0.08	1.67 ± 0.25	6.81 ± 0.11	0.82 ± 0.08	12.68 ± 0.32	1.81 ± 0.05	2.10 ± 0.07	0.21 ± 0.07	0.44 ± 0.05	0.45 ± 0.09

30	1.11 ± 0.39	1.96 ± 0.14	8.32 ± 1.05	1.02 ± 0.06	15.99 ± 0.84	2.15 ± 0.09	2.36 ± 0.07	0.25 ± 0.09	0.59 ± 0.05	0.61 ± 0.03
60	1.34 ± 0.23	2.87 ± 0.43	9.03 ± 1.23	1.57 ± 0.05	17.63 ± 0.32	2.51 ± 0.10	2.49 ± 0.12	0.30 ± 0.07	0.37 ± 0.03	0.79 ± 0.01
120	1.55 ± 0.21	1.81 ± 0.29	11.33 ± 1.43	1.53 ± 0.17	18.91 ± 0.22	2.94 ± 0.06	3.26 ± 0.02	0.25 ± 0.09	0.30 ± 0.01	0.86 ± 0.02
720	1.77 ± 0.35	1.01 ± 0.12	12.90 ± 2.22	0.93 ± 0.09	23.94 ± 0.71	1.70 ± 0.29	0.36 ± 0.07	0.13 ± 0.05	0.10 ± 0.02	0.22 ± 0

* Gallic acid (GA), (+)-Gallocatechin (GC), (-)-Epigallocatechin (EGC), Cianidanol (C), Caffeine (CAF), L-Epicatechin (EC), (-)-Epigallocatechin gallate (EGCG), (-)-Gallocatechin gallate (GCG), (-)-Epicatechin gallate (ECG), Catechin gallate (CG)

** Mean (± SD), n = 3

***ND, not detected, below the detection limit shown in Table 2.
